# Molecular and Metabolic Phenotyping of Hepatocellular Carcinoma for Biomarker Discovery: A Meta-Analysis

**DOI:** 10.3390/metabo13111112

**Published:** 2023-10-27

**Authors:** Nguyen Hoang Anh, Nguyen Phuoc Long, Young Jin Min, Yujin Ki, Sun Jo Kim, Cheol Woon Jung, Seongoh Park, Sung Won Kwon, Seul Ji Lee

**Affiliations:** 1 College of Pharmacy, Seoul National University, Seoul 08826, Republic of Korea; 2018-23140@snu.ac.kr (N.H.A.); m95511@snu.ac.kr (Y.J.M.); danielkim27@snu.ac.kr (S.J.K.); jungcw4906@snu.ac.kr (C.W.J.); swkwon@snu.ac.kr (S.W.K.); 2 Department of Pharmacology and PharmacoGenomics Research Center, Inje University College of Medicine, Busan 47392, Republic of Korea; 3 School of Mathematics, Statistics and Data Science, Sungshin Women’s University, Seoul 08826, Republic of Korea; 20160967@sungshin.ac.kr (Y.K.); spark6@sungshin.ac.kr (S.P.); 4 College of Pharmacy, Kangwon National University, Chuncheon 24341, Republic of Korea

**Keywords:** hepatocellular carcinoma, lipidomics, metabolomics, transcriptomics, meta-analysis

## Abstract

Identifying and translating hepatocellular carcinoma (HCC) biomarkers from bench to bedside using mass spectrometry-based metabolomics and lipidomics is hampered by inconsistent findings. Here, we investigated HCC at systemic and metabolism-centric multiomics levels by conducting a meta-analysis of quantitative evidence from 68 cohorts. Blood transcript biomarkers linked to the HCC metabolic phenotype were externally validated and prioritized. In the studies under investigation, about 600 metabolites were reported as putative HCC-associated biomarkers; 39, 20, and 10 metabolites and 52, 12, and 12 lipids were reported in three or more studies in HCC vs. Control, HCC vs. liver cirrhosis (LC), and LC vs. Control groups, respectively. Amino acids, fatty acids (increased 18:1), bile acids, and lysophosphatidylcholine were the most frequently reported biomarkers in HCC. *BAX* and *RAC1* showed a good correlation and were associated with poor prognosis. Our study proposes robust HCC biomarkers across diverse cohorts using a data-driven knowledge-based approach that is versatile and affordable for studying other diseases.

## 1. Introduction

Hepatocellular carcinoma (HCC) accounts for 90% of all liver cancers and is among the leading causes of cancer-related mortality worldwide [1,2]. Infection with hepatitis B and C viruses and alcoholic and nonalcoholic steatohepatitis are the leading risk factors for HCC [3]. HCC pathogenesis is highly heterogeneous and depends on multifactorial etiologies. Further research is required to elucidate the pathophysiology and the main drivers of HCC [4]. Despite advancements in molecular characterization and drug targets for HCC, limited therapies have successfully translated into improved clinical management [5]. HCC treatment is not remarkably effective; only a limited number of patients with early diagnoses receive curative treatment. Likewise, there are limited options for patients with advanced diseases [6]. Therefore, understanding the mechanisms underlying disease progression and identifying diagnostic biomarkers are essential.

Although metabolic reprogramming is a hallmark of cancer, the detailed analysis of aberrant metabolism in HCC requires extensive work [7]. Certainly, metabolomics can reveal accurate and deep insights into cancer biology and metabolism. Furthermore, the number of studies that employ metabolomics for identifying cancer biomarkers has increased exponentially, demonstrating its potential in clinical application [8]. Plasma and serum are the most conveniently and least invasively acquired samples and are commonly employed to discover biomarkers for cancer screening, diagnosis, prognosis, and therapeutic response [9]. Nonetheless, highly inconsistent finding for HCC is a well-regarded obstacle that hamper the translation into clinical implementations. Study design and experimental procedures, such as sample collection and preparation and statistical analysis, contribute to these differences, along with the application of different analytical platforms [10]. Essential information needed for accurately interpreting the results, including fasting conditions, quality control, and precise *p*-values, is often missing, furthermore the inconsistency and heterogeneity of the findings, resulting from the lack of standardization in clinical metabolomics, hamper high-quality meta-analysis in clinical metabolomic and lipidomic studies. Hence, a comprehensive and multimodal data mining approach is required to obtain insights into the molecular biology and metabolism of HCC and select biomarker candidates.

In this study, we investigated systemic and metabolism-centric multiomics levels of biologically relevant processes involved in HCC. A comprehensive search was conducted to gather all appropriate studies, and quantitative evidence of reported metabolites and lipids was provided. We further partially validated metabolite and gene biosignatures that could support the diagnosis of HCC. Our findings could provide insights into clinically relevant diagnosis as a more direct and practical approach to hopefully reducing the HCC burden.

## 2. Materials and Methods

### 2.1. Literature Search

We conducted a systematic search using three databases: PubMed, EMBASE, and Web of Science. A systematic review and meta-analysis were conducted following PRISMA 2020 guidelines (Supplementary File S1) [11]. The following queries were used to retrieve data from the databases: “(liver OR hepatocellular) AND (tumor OR tumor OR malignancy OR neoplasm OR cancer OR carcinoma OR adenoma) AND (“metabolite profiling” OR “metabolite analysis” OR “metabolic profiling” OR “metabolic fingerprinting” OR “metabolic characterization” OR metabolome OR metabolomics OR lipidome OR lipidomics OR lipidemia) AND (blood OR plasma OR serum)”. There was no restriction in the search period. The data were first obtained in September 2021 and updated periodically until January 2022.

### 2.2. Data Selection

The data gathered from all the databases were combined, and duplicates were removed. All records were used for title and abstract screening. Consideration of an article for full-text reading was based on the following: (1) clinical study involving blood (serum or plasma) metabolite or lipid profiling; (2) comparison of two of three groups: healthy control (Control), liver cirrhosis (LC), and HCC; and (3) inclusion of a mass spectrometry (MS)—based method or nuclear magnetic resonance (NMR) spectroscopy. The following exclusion criteria were used: (1) nonclinical study; (2) irrelevant control group; (3) specimens other than serum or plasma; (4) duplicated or parts of a more extensive study; and (5) review, abstract, case report, or conference papers. The qualified articles were collected for full-text evaluation and extraction of relevant data. At least two independent authors screened all the data. Any conflict was discussed and resolved by a third author via discussion or consensus.

### 2.3. Data Extraction

We extracted the study design and population characteristics (cohort allocation, sample size, sex, viral status, tumor stage, and reference diagnostic method). Next, the information on sample type (serum or plasma), instrument platform (LC−MS, GC−MS, and NMR), study procedure (sample preparation, sample storage, and internal standard), and statistical analysis (univariate, multivariate, and *p*-value adjustment methods) was extracted. Concerning metabolites and lipid reporting, the *p*-value, fold change, and expression profiles in HCC were recorded when applicable. Two independent authors extracted the data and discussed any conflicts to conclude.

### 2.4. Metabolite and Lipid Identification

Reported compounds were classified into either “metabolite” or “lipid,” owing to the differences in the subsequent analyses. In addition, the abbreviated notation was used for fatty acid (FA) and the conjugated and acetylcarnitine classes. Different metabolite synonyms were combined into one common name according to the Human Metabolome Database (HMDB) identification. If the HMDB ID was absent, the PubChem ID was used instead. LipidLynxX was used to convert all lipid compounds to lipid species level for further analysis because the reported practice of lipid nomenclature was inconsistent [12]. Regarding quality risk assessment, there is no generally approved risk assessment in omics research, particularly metabolomics and lipidomics in this work. Furthermore, we used a vote-counting method that did not incorporate statistical data from the included studies (e.g., mean, standard deviation). Therefore, in this study, the quality assessment step was skipped. Instead, study characteristics that could potentially affect study results were extracted and summarized to ensure the reproducibility of future studies.

### 2.5. Transcriptomics Meta-Analysis

Two peripheral blood mononuclear cell (PBMC) transcriptomic datasets comparing HCC vs. Controls—GSE49515 [13] and GSE58208—were retrieved from the Gene Expression Omnibus (GEO) repository. An individual dataset was processed and normalized using affy and lima packages before conducting a meta-analysis on NetworkAnalyst version 3.0 [14]. The Probe ID was mapped to the Entrez ID for nomenclature. Batch effect removal was applied using the Combat algorithm. A combined effect size with the random effects method was used to conduct a transcriptomic meta-analysis. GEO2R was used to analyze PBMC transcriptomics between cirrhosis and HCC (GSE10459) [15].

### 2.6. Pathway Enrichment Analysis

Pathway enrichment analysis was conducted to reveal the involvement of the molecular processes in HCC. Specifically, metabolites or lipids reported in at least two studies with vote counting different from zero were examined against the Kyoto Encyclopedia of Genes and Genomes (KEGG) database using MetaboAnalyst 5.0 [16]. Bile acids (Bas) and their derivatives were integrated with other metabolites, as they are curated well in KEGG. Three metabolite lists (HCC vs. Control, HCC vs. LC, and LC vs. Control) were analyzed separately.

The protein–protein interaction (PPI) network was used to analyze the PBMC transcriptomic data to explore the biological pathways associated with cancer progression. In particular, the blood tissue-specific module in NetworkAnalyst 3.0 was used to create the PPI network from a list of differentially expressed genes (DEGs).

### 2.7. Gene–Metabolite Interaction Network and Lipid-Related Gene Network

Gene–metabolite interaction network (GMIN) analysis was performed for two comparisons, HCC vs. Control and HCC vs. cirrhosis, using the Network Analysis module in MetaboAnalyst 5.0. In brief, lists of significant metabolites defined in the previous sections and significant genes were inputted to construct the gene–metabolite pathway related to HCC. Pathway analysis was subsequently conducted using the KEGG database. Significant lipid classes in the lipid class enrichment analysis were inputted to generate the lipid-related gene network using Lipidsig [17]. All genes related to the significant pathway were then overlapped with a significant gene list of corresponding comparisons and were considered potential biomarkers for disease progression.

### 2.8. Bioinformatics, Survival Analysis, Immunohistochemistry (IHC), and Machine Learning Model

The promising genes retrieved from gene–metabolite interaction and lipid-related gene network were further examined in liver transcriptomics. The survival analysis was also conducted to suggest prognosis biomarkers. In detail, RNA-sequencing (seq) data from The Cancer Genome Atlas (TCGA) [5] and the Genotype-Tissue Expression (GTEx) [18] databases were utilized to examine the expression of the biomarkers. Microarray data from the Oncopression database were also used [19]. IHC images of normal and cancer samples were retrieved from the Human Protein Atlas (https://www.proteinatlas.org, accessed on 16 February 2022) [20]. Overall survival analysis was conducted on GEPIA [21], Kaplan–Meier Plotter [22], and the Human Protein Atlas. The top candidates that show consistency in terms of gene expression and survival analysis among searched databases were selected for logistic modeling. In addition, we selected genes exhibiting upregulation in the cancer group with significant unfavorable survival. For logistic modeling, we first retrieved RNA-seq data from TCGA and GTEx via the TCGAbiolink package (last accessed on April 2022) [23] to fit a logistic regression model. A logistic model was fitted using the caret package. RNA-seq data for 371 HCC and 136 Control samples were divided into training and test subsets at a ratio of 7:3. This was repeated 10 times to avoid a specific pair of training/test data. The receiver operating characteristic (ROC) curve with the area under the curve (AUC) was used to evaluate the performance of each model. The mean and standard deviation (SD) of AUC from 10 datasets were determined.

### 2.9. Exploratory Data Analysis and Visualization

The vote-counting results were illustrated using the Amanida package. Heatmap visualization was performed using the GraphBio web app [24]. GMIN was run using MetaboAnalyst 5.0 and reconstructed using Gephi software version 0.9.2 [25]. The lipid-related gene network was visualized using the ggplot2 package [26]. The ROC curve was drawn using the pROC package [27].

### 2.10. Statistical Analysis

Vote-counting statistics were performed using the Amanida package [28]. In brief, a metabolite or lipid was regarded as “1” if reported as upregulated and “−1” if downregulated. The vote result was calculated based on the sum of the reported votes. Positive and negative vote results indicated overall upregulation and downregulation, respectively, whereas “0” indicated an equally reported number in both directions. We performed the sign test to determine whether this result occurred by chance. This is a nonparametric method requiring few assumptions for data and thus applies to general situations. It measures the possibility of vote results based on a binomial distribution and establishes how plausible it is to observe such results by chance. Metabolites and lipids reported in less than six studies were not tested owing to the lack of statistical power. A false discovery rate (FDR) was controlled using the Benjamini–Hochberg procedure. A *p*-value < 0.05 or FDR < 0.05 or <0.2 was considered to indicate statistical significance.

## 3. Results

### 3.1. Compendium Biomarker Report for HCC

**Figure 1:**
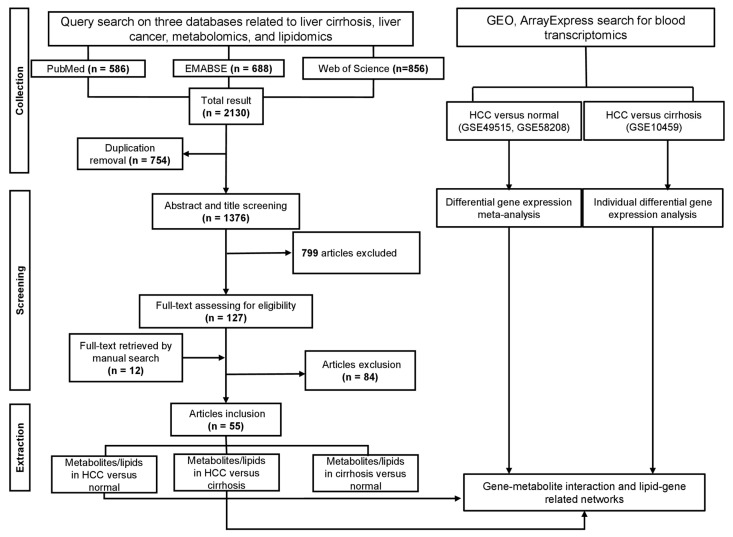
Workflow of this study. HCC: hepatocellular carcinoma, GEO: Gene Expression Omnibus.

A total of 55 studies involved 68 cohorts that consisted of an estimated 3325 patients with HCC, 2109 patients with LC, and 1693 healthy individuals (Control). Among them, 41 [29,30,31,32,33,34,35,36,37,38,39,40,41,42,43,44,45,46,47,48,49,50,51,52,53,54,55,56,57,58,59,60,61,62,63,64,65,66,67,68,69], 31 [29,30,31,32,33,34,35,36,37,38,39,40,41,42,43,44,45,46,47,48,70,71,72,73,74,75,76,77,78,79,80,81,82,83], and 14 [29,30,31,32,33,34,35,36,37,38,39,40,41,49] studies reported metabolic differences in HCC vs. Control, HCC vs. LC, and LC vs. Control groups, respectively. Many metabolomics and lipidomics datasets are not currently publicly available from data repositories. Therefore, we conducted a systematic search and comprehensive data synthesis of the currently reported blood metabolites and lipids in HCC. As a result, a compendium of metabolites and lipids in HCC with approximately 600 reported compounds was established. Figure 1 presents the workflow of this study.

**Figure 2:**
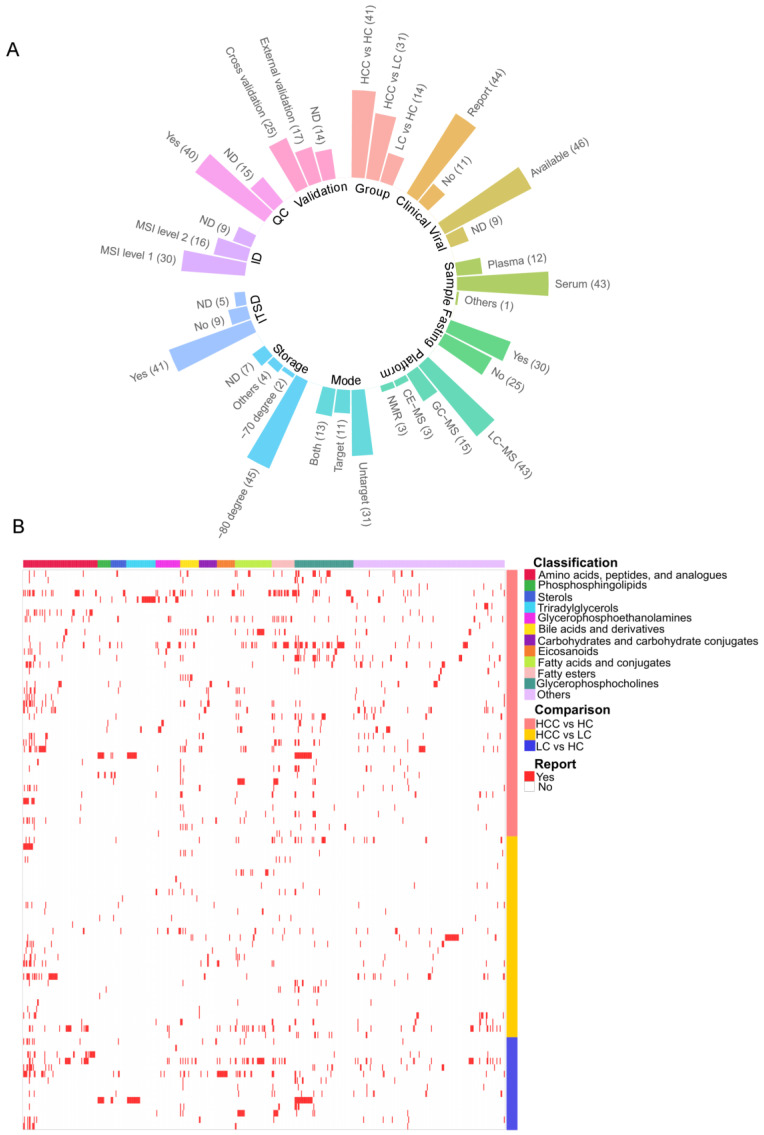
Study characteristics and overview of reported biomarkers in the included studies. (**A**) Characteristics of the included studies. (**B**) Metabolite and lipid reporting across the studies. HC: healthy control, HCC: hepatocellular carcinoma, LC: liver cirrhosis, MS: mass spectrometry, MSI: Metabolomics Standards Initiative, ND: not described, NMR: nuclear magnetic resonance.

Most studies were conducted in Asia (*n* = 40), followed by the United States (*n* = 9) and Europe (*n* = 6). In most studies, population characteristics were sufficiently reported (e.g., age, sex, and hepatitis B and/or hepatitis C viral status). Serum was primarily used for metabolic phenotyping and exploring metabolite and lipid differences (43 studies). In addition, only approximately half (*n* = 30) of the included studies involved sample collection in the fasting state; the fast/fed status was unknown in other studies. After sample collection, the samples were stored at −80 °C until their subsequent use in most studies. Notably, one study in which samples were stored at 4 °C was retained in our study, as we aimed to comprehensively reflect the current status of research. We noted that LC−MS was the most employed analytical platform (43 studies), followed by GC−MS (15 studies). An untargeted approach (*n* = 31) was commonly employed to characterize metabolite and lipid phenotypes over a targeted approach (*n* = 11). In addition, 13 studies used complementary untargeted and targeted analyses. The internal standard was used in 41 of the 55 studies for quality control and assessment purposes, whereas the use of pooled quality-control samples was described in 40 of the 55 studies. Figure 2 summarizes the key characteristics of the studies included in our investigation. More detailed information is presented in Table S1.

Metabolite reporting was classified based on the Metabolomics Standards Initiative (MSI) level. Interestingly, most included studies reported identification levels from level 1 to level 2. However, nine studies did not report the identification level. We noted that only 20 studies used the adjusted *p*-value to correct multiple hypotheses testing. The *p*-value was rarely reported beside the significance note (e.g., *p*-value < 0.05). The included studies spanned various demographics, etiologies, and instrument platforms, introducing a significant heterogeneity into the findings. The expression of approximately 600 molecules was significantly changed between stages of cancer progression (i.e., Control > LC > HCC) because cirrhosis is the most potent risk factor for HCC development. An overview of reported metabolites is shown in Figure 2B. The expression of 204, 144, and 105 metabolites was significantly altered (HCC vs. Control, HCC vs. LC, and LC vs. Control, respectively). The expression of amino acids, peptides, and analogs was the most commonly altered, following carbohydrates and carbohydrate conjugates. With respect to lipids, the expression of 274, 116, and 138 lipids was significantly altered in HCC vs. Control, HCC vs. LC, and LC vs. Control, respectively. BAs, FAs, and glycerophosphocholines (GPCs) were the most common altered lipid classes. Table S2 shows the full list of metabolites reported in each study. Notably, as aforementioned in the methods section, we did not conduct assessments of risk of bias for each included study because the appropriate tool is unavailable to metabolomics research.

### 3.2. Vote-Counting Meta-Analysis for Robust Reported Compounds

A robust meta-analysis of metabolomics and lipidomics was essential owing to the heterogeneity and lack of consistency in reporting the study results. With respect to the metabolites reported in HCC (compared with those in Control), we observed that the expression of L-tyrosine was mainly upregulated in patients with HCC when using vote-counting analysis (VCS = 10/13). In addition, L-phenylalanine and L-glutamic acid showed a high frequency of upregulation (VCS = 10/14 and VCS = 5/7, respectively). On the contrary, the levels of L-tryptophan seemed to decrease in patients with HCC (VCS = 6/7). In comparison with LC group, L-Glutamic acid was the metabolite with the highest reported levels, and the sign test revealed that the expression of L-glutamic acid was significantly upregulated in patients with HCC(VCS = 7/7, FDR = 0.03). We observed that the levels of L-valine (VCS = 5/5) and L-serine (VCS = 4/5) were primarily upregulated. In contrast, the expression of L-glutamic acid (VCS = 4/5, *p*-value = 0.19) was more substantially downregulated in LC than in Control. Overall, amino acids were the most reported metabolites. In particular, L-tyrosine, L-phenylalanine, L-glutamine, and L-glutamic acid seem to be the most robustly validated biomarkers across studies for HCC.

Lipids were reported more frequently than hydrophilic metabolites. BAs, lysoPCs (LPCs), and FAs were reported in many studies. Vote counting revealed that the levels of glycocholic acid (GCA) (VCS = 14/15, FDR = 0.007) and glycochenodeoxycholic acid (GCDCA) (VCS = 9/9, FDR = 0.004) increased consistently, whereas those of taurocholic acid (TCA) (VCS = 6/6, FDR = 0.034) decreased in HCC vs. Control. Interestingly, we observed a consistent downregulation with a statistically significant trend of LPC in HCC vs. Control, including LPC (16:0) (VCS = 12/12, FDR = 0.005), LPC (18:2) (VCS = 11/12, FDR = 0.012), and LPC (18:0) (VCS = 11/11, FDR = 0.004). Similarly, HCC vs. LC also revealed a dominant disturbance in the expression of BA, FA, and LPC classes. For instance, FA (18:2) (VCS = 5/6) and FA (18:1) (VCS = 4/4) exhibited increased levels, whereas GCA (VCS = 4/4) and LPC (18:2) (VCS = 4/4) showed the opposite trend.

Finally, in agreement with the results of the prior comparison, the alterations observed in lipidomics between LC and Control were at the level of BAs, FAs, and LPCs. The levels of GCDCA and GCA (both VCS = 4/4) showed an uptrend whereas those of LPC (16:0), LPC (18:0), LPC (18:2), and LPC (22:6) (all VCS = 3/3) showed a downtrend. Notably, FAs and BAs showed a discontinuous trend across HCC progression compared with LPCs. The levels of most BAs increased in HCC compared with those in Control but decreased compared with those in LC. Contrastingly, the levels of most LPC species consistently decreased from Control to LC toward HCC. The most robust candidates across studies included LPC (18:2), LPC (14:0), LPC (20:3), and LPC (20:5), which potentially serve as lipid biomarkers for HCC progression. The vote-counting results of the most robust compounds between each comparison are summarized in Table 1. The complete vote-counting results and the sign test results (when applicable) are presented in Table S3.

We next conducted pathway analysis on the commonly reported metabolites to obtain biologically meaningful insights. Based on this analysis, eight common significant pathways across the comparisons were identified. “Aminoacyl-tRNA biosynthesis”, “alanine, aspartate, and glutamate metabolism”, “phenylalanine, tyrosine, and tryptophan biosynthesis”, and “primary bile acid biosynthesis” were the notable pathways commonly shared among the comparisons. The results of pathway enrichment analysis are shown in Table 2 and Table S4A, Figure S1. With respect to lipid subclass enrichment, LPC, PC, and FAs and conjugates were the typical lipid classes altered among the comparisons. The result of lipid subclass enrichment analysis is presented in Table S4B.

### 3.3. Association of Blood Transcriptomics with HCC Pathogenesis

**Figure 3:**
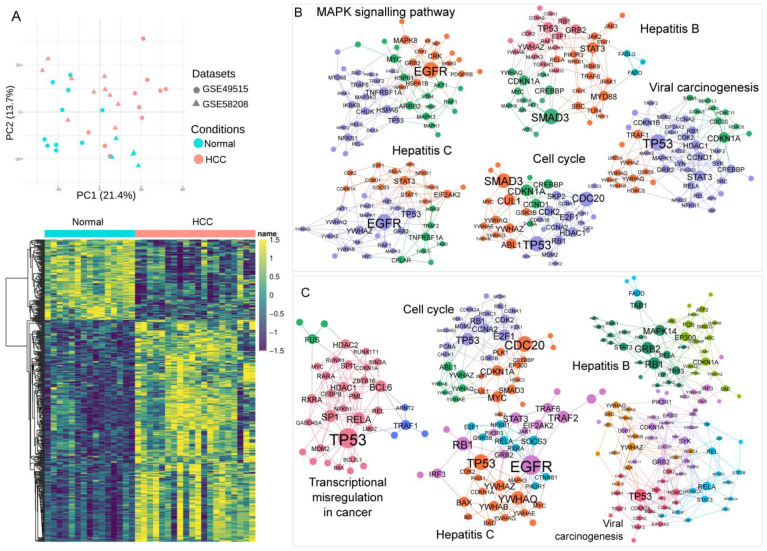
Results of blood transcriptomics analysis. (**A**) Principal component analysis and heatmap of the transcriptomics meta-analysis from two datasets: GSE49515 and GSE58208. (**B**) Blood-specific protein–protein interaction (PPI) network of differentially expressed genes (DEGs) from transcriptomics meta-analysis of hepatocellular carcinoma (HCC) vs. Control. Whole-blood PPI network was generated, and five representative PPI networks are presented. (**C**) Blood-specific PPI network of DEGs from single transcriptomics analysis of HCC vs. liver cirrhosis. Whole-blood PPI network was generated, and five representative PPI networks are presented.

Principal component analysis showed an apparent separation between samples in the HCC group and Control (Figure 3A). Combined effect-size meta-analysis revealed that the expression of 436 DEGs significantly changed in HCC vs. Control (Figure 3A and Table S5A). The blood–specific protein interaction network revealed pathways associated with hepatocarcinogenesis-related aspects, including viral carcinogenesis, hepatitis B, hepatitis C, and cell cycle (Figure 3B).

Likewise, using GEO2R to analyze single transcriptomics, we retrieved 482 DEGs from the cohort comparing HCC and LC. Interestingly, the blood-specific PPI pathway was also linked to cancer pathogenesis. Many pathways overlapped with those in the PPI network of HCC vs. Control, such as hepatitis B, hepatitis C, viral carcinogenesis, and cell cycle (Figure 3C). Blood transcriptomics suggested a connection between PBMCs and HCC. The full DEG list for each comparison is in Table S5. Table S6 lists all the pathways from the blood-specific PPI analysis.

### 3.4. Gene–Metabolite Network Analysis and Lipid-Related Gene Network

Next, a GMIN was constructed. Only HCC vs. Control and HCC vs. LC comparisons were subjected to the analysis. GMIN revealed the interplay among highly reported metabolites and DEGs. With respect to HCC vs. Control, the metabolites identified to play a central role using GMIN were primarily amino acids, such as l-glutamic acid, l-tyrosine, l-phenylalanine, and l-glutamine.

**Figure 4:**
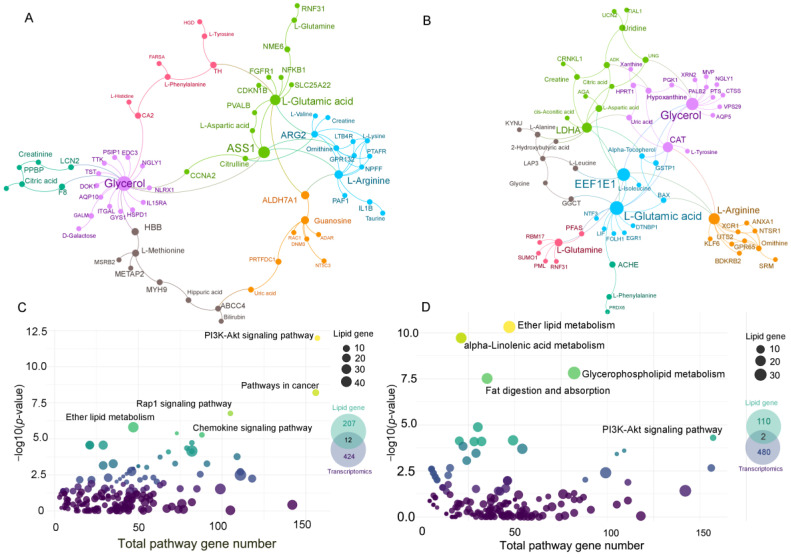
Gene–metabolite interaction network (GMIN) and lipid-related gene networks from two comparisons: hepatocellular carcinoma (HCC) vs. Control and HCC vs. liver cirrhosis. (**A**) GMIN of HCC vs. Control comparison. (**B**) HCC vs. Control and HCC vs. liver cirrhosis. Color was assigned based on the modular similarity calculation. (**C**) Lipid-related gene network of HCC vs. Control. (**D**) Lipid-related gene network of HCC vs. liver cirrhosis. The human KEGG pathway was suggested based on the input of lipid classes; lipid genes belonging to significant pathways were overlapped with differentially expressed genes to find common genes.

Under HCC vs. Control, metabolites presented in the GMIN of HCC vs. LC were frequently reported. Genes and metabolites interacting in GMIN are intermediaries of various pathways, including “arginine biosynthesis”, “aminoacyl-tRNA biosynthesis pathway”, and “alanine, aspartate, and glutamate metabolism”. These pathways were also found to be significant in previous metabolite pathway enrichment analyses. In GMIN, glutamine, glutamate, glycerol, and arginine were hub-interacting metabolites, whereas *ASS1*, *AGR2*, *LDHA*, and *EEF1E1* were the most interacting genes in the networks. The consistent results indicate the potential role of these genes and metabolites in HCC progression. An overview of GMIN for two comparisons is presented in Figure 4A,B, and GMIN pathway enrichments and potential genes and metabolites in the networks are shown in Table S7.

In terms of the lipid-related gene network, several pathways related to cancer-altered signaling and metabolism were found, including “PI3K-Akt signaling pathway”, “ether lipid metabolism”, and “alpha-linolenic acid metabolism”. Among gene-related lipid metabolism pathways, we found 12 and 2 genes that overlapped with the DEGs from HCC vs. Control and HCC vs. LC, respectively. More importantly, a group of genes encoded enzymes for phospholipid metabolism, particularly LPC. These genes might explain the behavior of LPC in our study report. The details of the lipid-related gene pathway are shown in Figure 4C,D and Table S8.

### 3.5. Bioinformatic Analysis and Prediction Model

**Figure 5:**
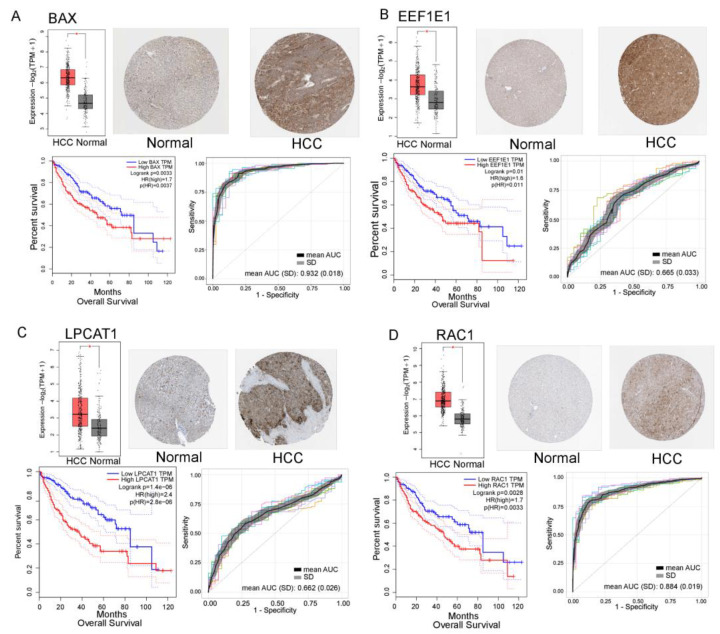
Bioinformatic analysis of potential protein biomarkers derived from the gene–metabolite interaction network and the lipid-related gene network. (**A**) BAX. (**B**) EEF1E1. (**C**) LPCAT1. (**D**) RAC1. RNA-seq data from The Cancer Genome Atlas (TCGA) and Genotype-Tissue Expression (GTEx) databases were retrieved to evaluate the expression levels of the candidates. The box plot represents the RNA-seq expression of each candidate in hepatocellular carcinoma (HCC) compared with matched TCGA normal and GTEx data; immunohistochemistry image for each candidate was retrieved from The Human Proteome Atlas. The overall survival Kaplan–Meier plot of each candidate was examined with a median cutoff. The receiver operating characteristic (ROC) curve of the logistic regression model was built using the TCGA-GTEx combined RNA-seq data with 10 times repeated data splitting (7:3); mean area under the curve (AUC) and SD values across 10 models are presented. However, only the first five ROC curves are presented in the graph, colors indicate logistic regression models * Indicates significance with FDR < 0.05. SD: standard deviation.

Genes involved in GMIN and lipid-related gene network were further examined in the tumor. mRNA expression, protein expression, and prognosis of the most potential candidates were analyzed using data from TCGA, Human Protein Atlas, GEPIA, and Oncopression. Many genes were significantly and differentially expressed or significantly associated with prognosis and high/low protein expression in HCC tissue compared with that in Control. *BAX*, *EEF1E1*, *LPCAT1*, and *RAC1* showed upregulation in expression and were also associated with poor prognosis in HCC. These four genes were highly differentially expressed in HCC compared with those in the control group (TCGA-GTEx RNA-seq and Oncopression). In addition, their expression investigated using IHC tended to be higher in HCC than in Control. Overall survival analysis showed that patients with HCC who showed higher expression of these genes had significantly shorter survival periods than patients who showed lower expression. *BAX*, *EEF1E1*, *LPCAT1*, and *RAC1* appear to play an essential role in HCC pathophysiology. Detailed information for all potential genes is presented in Figure 5 and Table S9.

Furthermore, to evaluate the diagnostic ability of these markers, we built a logistic regression model using combined TCGA and GTEx transcriptomic data. The RNA-seq data available was for 371 tumors and 136 controls. Then, samples were divided at 7:3 for a training and test set, and the analysis was repeated 10 times. Interestingly, *BAX* and *RAC1* showed promising performance with respect to distinguishing between HCC and Control. The mean AUC of *BAX* and *RAC1* was 0.932 and 0.884, whereas that for *EEF1E1* and *LPCAT1* was 0.665 and 0.662, respectively (Figure 5). A model combining these four genes achieved satisfactory accuracy, with a mean AUC of 0.950 (Figure S2). Evidence indicates that *BAX* and *RAC1* can serve as promising biomarkers for HCC progression, diagnosis, and prognosis.

## 4. Discussion

We performed an integrated meta-analysis on metabolomic, lipidomic, and transcriptomic data to explore biomarkers for supporting the detection, diagnosis, and prognosis of HCC. Overall, quantitative evidence from 55 metabolomic studies was synthesized via a data-driven and knowledge-based framework, followed by an additional blood transcriptome analysis to identify promising transcript biomarkers. This integrated approach provides new avenues to explore the interactions between genes and metabolites, HCC-related biological pathways, and novel biomarkers.

Metabolic alteration has been considered a hallmark of cancer, including HCC. The development of omics sciences and technologies has enabled the exploration of genetic variants, alterations in the expression of functional molecules, and their dynamic interactions with respect to cancer pathogenesis. Despite the tremendous success of metabolomics in translational and clinical cancer research, standardization of instrumental and data analyses remains unavailable. This, in addition to the diverse demographic and genetic background of the studied population, subsequently resulted in significant heterogeneity and inconsistent reporting across published studies.

Approximately six hundred disparate metabolites were reported in the studies under investigation. This inconsistency can be explained by the diverse population characteristics, quality control, sample handling, data acquisition, statistical methods, and metabolite identification. The inconsistency in biomarker reporting has been emphasized in various systematic reviews that consider metabolomics [84]. While time and effort are needed to standardize metabolomic and lipidomic research, a systematic evidence synthesis study could provide considerable advantages with respect to facilitating the next phase of biomarker discovery and validation.

Among the 600 molecules, only 39, 20, and 10 metabolites and 52, 12, and 12 lipids were reported in three or more studies in HCC vs. Control, HCC vs. LC, and LC vs. Control, respectively. Inconsistency in molecular behavior was observed among comparisons. The levels of amino acids (e.g., L-phenylalanine and L-glutamic acid), FAs (e.g., FA (18:1) and FA (18:2)), BAs (e.g., GCA, GCDA, and TCA), and GPCs (e.g., several LPC and PC species) were primarily altered during HCC progression and may be considered the most promising biomarker candidates. At least two studies showed that the levels of glucose and glutamine, the primary fuel sources in cancer cells, were robustly reduced in HCC vs. LC or Control. A consistent elevation in glutamate levels has been reported in studies on the progression from LC to HCC. Alone or combined with alpha-fetoprotein, metabolite and lipid signatures can serve as a powerful approach for screening cancer progression and timely diagnosis. These metabolites and lipids can serve as potential biomarkers for each stage of disease progression. Deep insights into these metabolic alterations may also provide novel therapeutic targets.

Amino acid metabolism and biosynthesis, including branched-chain amino acids (BCAAs, e.g., leucine, isoleucine, and valine), aromatic amino acids (AAAs, e.g., tyrosine, phenylalanine, and tryptophan), and arginine, were enriched. BCAAs are nitrogen donors and important nutrient sources, which can buttress intrinsic cancer properties and partially reflect the systemic metabolism alteration in cancer [85]. Unlike those of BCAAs, the functions of AAAs in cancer have not been comprehensively investigated yet. However, a recent study revealed tryptophan-to-phenylalanine substitutions to be associated with cancers that emerge under conditions of amino acid deprivation [86]. The effects of arginine depletion therapy on numerous cancer types have been investigated in phase I clinical trials [87]. Identification of systemic blood amino acid components could benefit biomarker discovery or cancer therapy.

BAs are important steroids synthesized in the liver and play a crucial role in immune responses, apoptosis, and glucose metabolism. Many studies reported decreased BA levels when comparing HCC to LC. The excessive accumulation of BA results in hepatocyte necrosis and apoptosis. Existing evidence indicates that the levels of primary BAs significantly increased in cirrhosis and HCC, with the highest level appearing at the cirrhosis stage. The elevation of GCDCA and taurochenodeoxycholic acid levels induced hepatocyte damage, hepatoxicity, and apoptosis. Some studies revealed that BAs are involved in the pathology of nonalcoholic fatty liver disease and nonalcoholic steatohepatitis that can progress to HCC [88,89]. The regulatory function of BAs with respect to the gut microbiome and immune responses has recently been reported. Likewise, prediagnostic serum-conjugated primary BAs were promising HCC surveillance and diagnostic markers in a study with over 200 samples per group [90]. Another report showed that the suppression of BA synthesis aids hepatocarcinogenesis in mice [91].

Furthermore, FAs are one of the main lipid classes altered during HCC progression in our study. In most cases, FAs were continuously upregulated during liver cancer progression. De novo synthesis of FAs has been well documented to be associated with tumor growth. The alterations in the levels of FAs could be used as biosignatures for HCC. Indeed, inflammation resulting in metabolic disturbance is a key phenomenon in HCC, with FA homeostasis playing a crucial role. Previous studies have linked aberrant FA transport to HCC progression and metastasis [92,93]. The levels of plasma membrane lipids, particularly LPC and PC, are robustly and consistently decreased during HCC progression. Recent reviews showed that membrane phospholipids work in concert with other lipids and promote *EGFR* clustering at the plasma membrane, contributing to oncogenic signaling [94]. Oxidative stress is now considered one of the factors aiding HCC progression [3]. The progression from LC to HCC is difficult to distinguish, resulting in late-stage diagnosis and a high mortality rate. Evidence indicates that BAs, FAs, and LPCs can serve as potential biomarkers for HCC progression.

Mutant genes associated with HCC evolution interacted with DEGs, such as *TP53*, *MYC*, *EGFR*, and *CTNNB1*. The expression of RB1, another well-known HCC gene, was elevated in HCC vs. LC. Finally, several DEGs with high degrees of interaction in the PPI network were also associated with the pathophysiology of HCC. For instance, one study reported that *CUL3* deficiency altered the tumor microenvironment and induced cholangiocarcinoma development [95]. Additionally, knockout of *CAND1*, a regulator of Cullin–RING ubiquitin ligases, suppressed liver cancer cell proliferation by activating apoptosis [96].

Many genes are associated with downstream metabolism, particularly metabolic regulator genes. The expression of enzyme-coding genes, such as *LPCAT* in glycerophospholipid metabolism and *ELOVL7* in fatty acid elongation, was upregulated. In contrast, ALDH7A1 in lysine degradation was downregulated in HCC vs. Control. The expression of *LPIN3* in glycerophospholipid metabolism was upregulated, while that of *CAT* in tryptophan metabolism and glyoxylate and dicarboxylate metabolism was downregulated in HCC vs. LC. Gene–metabolite analysis provided hints that may benefit subsequent mechanistic studies. Our GMIN analysis found that many metabolism-related DEGs widely interact with commonly reported metabolites. For instance, glutamic acid and glycerol were central nodes interacting with oncogenic or metabolic genes in both comparisons (HCC vs. LC and HCC vs. HC). Glutamic acid interacted with several genes related to HCC, including *CDKN1B* [97] and *ARG2* [98] in HCC vs. LC and *EEF1E1* [99] and *LDHA* [100] in HCC vs. Control. Further, glycerol, the backbone of glycerophospholipid, plays a central role in GMIN, which may explain why LPC and PC were dominantly reported across studies. Other worth-mentioning genes and metabolites previously reported in cancer progression are *ASS1*, *CAT*, *ALDH7A1*, arginine, glutamine, BCAAs, and AAAs. These hub genes/metabolites may represent the systemic alterations of HCC metabolism and partially provide evidence for the association of complementary gene and metabolite dysregulations in HCC. Except for BA, most typical compounds originate from the liver. Most of the metabolites interacting in GMIN were consistently reported among the included studies. This again confirms the robustness and potential usability of these candidates as biomarkers for HCC. Collectively, GMIN helps to explain the phenome alterations of the disease. It also facilitates biomarker candidate prioritization and opens new avenues for developing integrated assays aimed at detecting disease or predicting disease progression.

With respect to the lipid function, as we are far from constructing lipid pathways, our approach connects the enrichment lipid class with lipid-related gene network. Interestingly, the observed lipid-related gene pathways are also linked to cancer. For instance, the PI3K-Akt signaling pathway is a well-known cascade in hepatocarcinogenesis [101], whereas the Rap1 signaling pathway regulates hematopoietic stem cell survival and therapeutic response of breast and colon cancer [102]. This suggests that lipid class systemic alteration may reflect the metabolic signature of a specific cancer.

*RAC1*, *BAX*, *EEF1E1*, and *LPCAT1*, four highly interacted genes, showed differential expression at gene levels in HCC and played essential roles in cancer cells. *EEF1E1* was recently mentioned as an independent prognostic factor in HCC and was correlated with the tumor immune microenvironment [99]. *LPCAT1* has been linked with the progression of various types of cancer via the reprogramming of cholesterol metabolism [103] or regulating oncogenic signaling [104]. It has also been reported to be involved in the proliferation, invasion, and migration of HCC cells [105]. Further, active expression of *RAC1* was usually linked to the promotion of metastasis and drug resistance, and it was considered a targeted cancer therapy in melanoma [106] and breast cancer [107]. Finally, *BAX* is a critical effector of mitochondrial apoptosis linked to apoptosis-resistant cancer cells [108]. The upregulated expression of these four genes was strongly correlated with poor outcomes in HCC and other cancer types. Mainly, *BAX* and *RAC1* could serve as surveillance and prognostic biomarkers and therapeutic targets.

Our study provides comprehensive quantitative evidence regarding the association of blood metabolites and lipids with HCC progression. A proper statistical approach was used to detect significant trends and suggested amino acids, BAs, and FA levels were associated with HCC progression. Furthermore, the integrated meta-analysis approach provided mechanistic insights into the gene–metabolite interaction network and the lipid-related gene network in HCC. However, there are several limitations to our approach. Only a few metabolites could be statistically analyzed due to the limitation of the number of studies used for analysis. In addition, studies with a relatively small sample size might lead to a high rate of false positives, which may subsequently introduce bias into the vote-counting approach. Second, potential sources of heterogeneity, such as different analytical platforms, HCC etiology, study design, and gender, could lead to biased evidence summarization and require further validation. Notably, we did not conduct quality assessment in this study because there is no standardized quality assessment tool for metabolomic study. In addition, most of the included studies were conducted in Asian. All of these issues could limit the generalization of the findings as HCC is highly heterogeneous at different levels. Therefore, subsequent studies are warranted to explore risk factors and ethnic differences in HCC fully. Finally, the protein expression and association to the prognosis of proposed candidates should also be further validated in independent cohorts to verify their clinical validity. Additionally, the vote-counting evidence purely presents the general behavior of metabolites and lipids associated to HCC. Therefore, a causative risk relationship should not be stated between the highly reported metabolites and liver function impairment. Our study proposed an approach to deriving meaningful biological insight and robust biomarkers by integrating quantitative evidence. To ensure quality of candidates, future confirmatory analysis is warranted to confirm the validity of the biomarkers with a large cohort. This approach is advantageous when more time is needed to standardize the metabolomic and lipidomic reports.

## 5. Conclusions

Heterogeneity and inconsistency in the findings of clinical metabolomics hamper the biomarker translation from the bench to the bedside. More effort is required to standardize the utilized methods and guarantee the reproducibility of the findings. In the meantime, prioritizing the biomarker candidates using the data-driven and knowledge-based framework is a practical solution. We suggest consistent biomarkers for HCC across a diversity of cohorts, platforms, and workflows via a quantitative synthesis approach and integrated meta-analysis. Our findings provide reasonably comprehensive blood metabolomics of HCC progression and identified genes, metabolites, and lipid biomarkers for HCC surveillance, diagnosis, and prognosis.

## Figures and Tables

**Table 1:** Vote-counting results of the most robust significantly reported metabolites and lipids.

Polar Metabolite/Lipid	Votes ^a^	Number of Articles	Vote Counting ^a^	FDR	Type
HCC versus HC					
L-phenylalanine	6	14	0.43	0.54	Metabolite
L-tyrosine	7	13	0.54	0.83	Metabolite
L-leucine	0	8	0.00	1.00	Metabolite
L-serine	0	8	0.00	1.00	Metabolite
L-tryptophan	−5	7	−0.71	0.56	Metabolite
L-glutamic acid	3	7	0.43	1.00	Metabolite
L-proline	3	7	0.43	0.82	Metabolite
Ornithine	2	6	0.33	1.00	Metabolite
Taurine	−2	6	−0.33	0.88	Metabolite
Creatine	−1	5	−0.20	NA	Metabolite
Creatinine	−3	5	−0.60	NA	Metabolite
L-alanine	1	5	0.20	NA	Metabolite
L-methionine	3	5	0.60	NA	Metabolite
Uric acid	1	5	0.20	NA	Metabolite
D-glucose	0	4	0.00	NA	Metabolite
Glycerol	−2	4	−0.50	NA	Metabolite
Hypoxanthine	0	4	0.00	NA	Metabolite
L-aspartic acid	−2	4	−0.50	NA	Metabolite
L-isoleucine	−2	4	−0.50	NA	Metabolite
L-valine	−2	4	−0.50	NA	Metabolite
Myo-inositol	−2	4	−0.50	NA	Metabolite
Oxoproline	0	4	0.00	NA	Metabolite
Phenylalanyl phenylalanine	−4	4	−1.00	NA	Metabolite
Uridine	0	4	0.00	NA	Metabolite
LPC (16:0)	−12	12	−1.00	0.005 ^b^	Lipid
Glycocholic acid	13	15	0.87	0.007 ^b^	Lipid
LPC (18:0)	−11	11	−1.00	0.004 ^b^	Lipid
Glycochenodeoxycholic acid	9	9	1.00	0.013 ^b^	Lipid
LPC (18:1)	−9	9	−1.00	0.010 ^b^	Lipid
LPC (20:4)	−9	9	−1.00	0.009 ^b^	Lipid
LPC (18:2)	−10	12	−0.83	0.012 ^b^	Lipid
LPC (14:0)	−8	8	−1.00	0.013 ^b^	Lipid
LPC (20:3)	−7	7	−1.00	0.023 ^b^	Lipid
LPC (20:5)	−7	7	−1.00	0.020 ^b^	Lipid
LPC (22:6)	−7	7	−1.00	0.018 ^b^	Lipid
Taurocholic acid	6	6	1.00	0.034 ^b^	Lipid
CAR (10:0)	−6	6	−1.00	0.031 ^b^	Lipid
CAR (18:1)	5	5	1.00	NA	Lipid
PC (32:1)	5	5	1.00	NA	Lipid
LPC (17:0)	−5	5	−1.00	NA	Lipid
PC (38:6)	−5	5	−1.00	NA	Lipid
FA (18:1)	4	6	0.67	NA	Lipid
FA (18:2)	4	6	0.67	NA	Lipid
CAR (2:0)	3	5	0.60	NA	Lipid
CAR (8:0)	−3	5	−0.60	NA	Lipid
FA (20:4)	0	8	0.00	NA	Lipid
CAR (16:1)	4	4	1.00	NA	Lipid
CAR (18:2)	4	4	1.00	NA	Lipid
FA (16:1)	4	4	1.00	NA	Lipid
PC (32:0)	4	4	1.00	NA	Lipid
Taurochenodesoxycholic acid	4	4	1.00	NA	Lipid
FA (22:6)	2	4	0.50	NA	Lipid
CAR (16:0)	0	4	0.00	NA	Lipid
Oleamide	0	4	0.00	NA	Lipid
PE (38:6)	0	4	0.00	NA	Lipid
FA (20:5)	−2	4	−0.50	NA	Lipid
LPC (15:0)	−4	4	−1.00	NA	Lipid
LPC (18:3)	−4	4	−1.00	NA	Lipid
HCC versus LC					
L-glutamic acid	7	7	1.00	0.0312 ^b^	Metabolite
L-phenylalanine	−1	7	−0.14	1	Metabolite
L-serine	3	5	0.60	NA	Metabolite
L-valine	5	5	1.00	NA	Metabolite
L-isoleucine	2	4	0.50	NA	Metabolite
L-methionine	0	4	0.00	NA	Metabolite
L-proline	0	4	0.00	NA	Metabolite
L-tyrosine	−2	4	−0.50	NA	Metabolite
1-methyladenosine	3	3	1.00	NA	Metabolite
2-hydroxybutyric acid	3	3	1.00	NA	Metabolite
Citric acid	−1	3	−0.33	NA	Metabolite
Creatine	1	3	0.33	NA	Metabolite
Glycerol	−1	3	−0.33	NA	Metabolite
Glycine	1	3	0.33	NA	Metabolite
Hypoxanthine	3	3	1.00	NA	Metabolite
L-alanine	3	3	1.00	NA	Metabolite
L-aspartic acid	3	3	1.00	NA	Metabolite
Ornithine	3	3	1.00	NA	Metabolite
Uric acid	−1	3	−0.33	NA	Metabolite
Xanthine	−1	3	−0.33	NA	Metabolite
FA (18:2)	4	6	0.67	NA	Lipid
LPC (18:0)	1	5	0.20	NA	Lipid
CAR (2:0)	2	4	0.50	NA	Lipid
FA (18:1)	4	4	1.00	NA	Lipid
Glycocholic acid	−4	4	−1.00	NA	Lipid
LPC (16:0)	2	4	0.50	NA	Lipid
LPC (18:2)	−4	4	−1.00	NA	Lipid
CAR (0:0)	3	3	1.00	NA	Lipid
CAR (18:1)	−1	3	−0.33	NA	Lipid
FA (18:3)	1	3	0.33	NA	Lipid
FA (20:4)	1	3	0.33	NA	Lipid
LPE (16:0)	1	3	0.33	NA	Lipid
LC versus HC					
L-phenylalanine	5	9	0.56	0.36	Metabolite
L-serine	0	6	0.00	1	Metabolite
L-tyrosine	4	6	0.67	NA	Metabolite
L-glutamic acid	−3	5	−0.60	NA	Metabolite
L-methionine	4	4	1.00	NA	Metabolite
Bilirubin	3	3	1.00	NA	Metabolite
Glycine	−1	3	−0.33	NA	Metabolite
L-aspartic acid	−1	3	−0.33	NA	Metabolite
L-proline	−1	3	−0.33	NA	Metabolite
Ornithine	3	3	1.00	NA	Metabolite
FA (18:2)	1	5	0.2	NA	Lipid
FA (20:4)	−1	5	−0.2	NA	Lipid
FA (18:0)	2	4	0.5	NA	Lipid
Glycochenodeoxycholic acid	4	4	1	NA	Lipid
Glycocholic acid	4	4	1	NA	Lipid
CAR (2:0)	3	3	1	NA	Lipid
FA (16:1)	3	3	1	NA	Lipid
FA (18:1)	3	3	1	NA	Lipid
LPC (16:0)	−3	3	−1	NA	Lipid
LPC (18:0)	−3	3	−1	NA	Lipid
LPC (18:2)	−3	3	−1	NA	Lipid
LPC (22:6)	−3	3	−1	NA	Lipid

Three comparisons were performed: hepatocellular carcinoma (HCC) vs. healthy control (HC), HCC vs. liver cirrhosis (LC), and LC vs. HC. ^a^ A positive value indicates the final upregulated direction of the reported metabolites/lipids, whereas a negative value indicates the final downregulated direction of the reported metabolites/lipids. ^b^ A significant trend by the two-sided sign test with FDR < 0.05. FA: fatty acid, LPC: lysophosphatidylcholine, LPE: lysophosphatidylethanolamine, PC: phosphatidylcholine, PE: phosphatidylethanolamine, CAR: acylcarnitine, NA: not applicable, FDR: false-discovery rate.

**Table 2:** Results of pathway enrichment analysis.

Pathway Name	Significantly Enriched Pathways ^a^		
	HCC vs. Control	HCC vs. LC	LC vs. Control
Alanine, aspartate, and glutamate metabolism	o	o	o
Aminoacyl-tRNA biosynthesis	o	o	o
Arginine and proline metabolism	o	o	o
Arginine biosynthesis	o	o	o
D-glutamine and D-glutamate metabolism	o	o	x
Glyoxylate and dicarboxylate metabolism	o	o	o
Nitrogen metabolism	o	o	x
Phenylalanine metabolism	o	o	o
Phenylalanine, tyrosine, and tryptophan biosynthesis	o	o	o
Primary bile acid biosynthesis	o	o	o
Valine, leucine, and isoleucine biosynthesis	o	o	x
Glutathione metabolism	x	o	o
Ascorbate and aldarate metabolism	o	x	x
Butanoate metabolism	o	x	o
Citrate cycle (TCA cycle)	o	x	o
Glycine, serine, and threonine metabolism	o	x	x
Histidine metabolism	o	x	o
Porphyrin and chlorophyll metabolism	o	x	o
Pyruvate metabolism	o	x	x
Taurine and hypotaurine metabolism	o	x	x

^a^ FDR < 0.2; HCC: hepatocellular carcinoma, LC: liver cirrhosis, o: significant enrichment, x: nonsignificant enrichment, TCA: taurocholic acid.

## Data Availability

No raw data was generated in this study. Available data in the literature and public repositories were used.

## Supplementary Materials

The following supporting information can be downloaded at: https://www.mdpi.com/article/10.3390/metabo13111112/s1, Figure S1: Metabolite enrichment analysis. A. Hepatocellular carcinoma (HCC) versus control (HC), B. HCC versus liver cirrhosis (LC), C. LC versus HC, D. Overlapping pathways from all comparisons; Figure S2: Receiver operating characteristic (ROC) curve of logistic regression model for four candidates (BAX, EEF1E1, RAC1, and LPCAT1). 1–5: ROCs of the first five logistic regression models. AUC: area under the curve; Table S1: Demographic, study design, and analytical approach characteristics of the included studies; Table S2: The characteristics of all reported compounds across the included studies; Table S3A: Vote counting result of HCC vs. Control; Table S3B: Vote counting result of HCC vs. LC; Table S3C: Vote counting result of LC vs. Control; Table S4A: Metabolite enrichment pathway analysis result; Table S4B: Lipid sub-class enrichment analysis result; Table S5A: Differential gene expression from the PBMC transcriptomics meta-analysis of hepatocellular carcinoma versus control; Table S5B: Differential gene expression from the PBMC transcriptomics of hepatocellular carcinoma versus liver cirrhosis; Table S6: The blood-specific protein interaction networks of differential expression gene from each comparison; Table S7A: Pathway enrichment of gene-metabolite interaction network; Table S7B: Potential hub genes/metabolite in gene-metabolite interaction network; Table S8: KEGG lipid-related gene networks result from lipid enrichment sub-class; Table S9: Bioinformatics of potential hub genes in hepatocellular carcinoma. Reference [11] has been cited in the Supplementary Materials.
